# Affective Contagion: How Attitudes Expressed by Others Influence Our Perception of Actions

**DOI:** 10.3389/fnhum.2021.712550

**Published:** 2021-08-30

**Authors:** Giuseppe Di Cesare, Annalisa Pelosi, Silvia Maria Aresta, Giada Lombardi, Alessandra Sciutti

**Affiliations:** ^1^Cognitive Architecture for Collaborative Technologies Unit, Istituto Italiano di Tecnologia, Genova, Italy; ^2^Department of Medicine and Surgery, Neuroscience Unit, University of Parma, Parma, Italy; ^3^Department of Informatics, Bioengineering, Robotics and Systems Engineering (DIBRIS), University of Genova, Genoa, Italy

**Keywords:** vitality forms, action perception, affective contagion, action planning, motor imagery, action style

## Abstract

Vitality forms represent a fundamental aspect of social interactions by characterizing *how* actions are performed and *how* words are pronounced on the basis of the attitude of the agent. Same action, such as a handshake, may have a different impact on the receiver when it is performed kindly or vigorously, and similarly, a gentle or rude tone of voice may have a different impact on the listener. In the present study, we carried out two experiments that aimed to investigate whether and how vocal requests conveying different vitality forms can influence the perception of goal-directed actions and to measure the duration of this effect over time. More specifically, participants were asked to listen to the voice of an actor pronouncing “give me” in a rude or gentle way. Then, they were asked to observe the initial part of a rude or a gentle passing action, continue it mentally, and estimate the time of its completion. Results showed that the perception of different vitality forms expressed by vocal requests influenced the estimation of action duration. Moreover, we found that this effect was limited to a certain time interval (800 ms), after which it started to decay.

## Introduction

The observation of goal-directed actions performed by another individual allows one to understand what, why, and how that individual is doing it. During social interactions, by observing how actions are performed or by listening to the tone of voice, people can understand the affective state of others. Indeed, actions and speech dynamics represent the fundamental aspects of social communication, defined as “*vitality forms*” by Daniel Stern (2010). Vitality forms characterize human behavior by enhancing the quality of interactions. In particular, the expression of vitality forms enables the agent to communicate their affective states, while the perception of vitality forms allows the receiver to capture the affective states of the agent immediately (Di Cesare et al., 2013). For example, a hand gesture can be performed vigorously or kindly, a tone of voice can be unpleasant or pleasant, suggesting that the agent has a negative or positive mood/attitude toward the receiver. It is important to note that vitality forms differ from basic emotions. According to Darwin (1859) and James (1884), basic emotions are short-lasting events characterized by visceromotor responses and preparation to act. In contrast, vitality forms represent the manner in which actions are performed and reflect the affective state of the agent, modulating his behavior in a continuous manner.

Several fMRI studies have investigated the neural correlates involved in the processing of vitality forms, showing that the dorsocentral insula has a crucial role in the perception, planning, and execution of actions conveying gentle and rude vitality forms (Di Cesare et al., 2015,2016a,2017b,2020a,2020b). In addition to actions, human interactions rely on linguistic exchanges. Indeed, Di Cesare et al. (2017b) demonstrated that listening to action verbs pronounced with gentle and rude vitality forms and imaging to pronounce the same action verbs with the same vitality forms activated the parietofrontal circuit and the dorsocentral sector of the insula (Di Cesare et al., 2016b). While the activation of the parietofrontal circuit indicated that individuals internally represented the listening/imaging actions communicated by verbs, the activation of the insula showed that they also relived the forms (gentle, rude) of those actions. Thus, the activation of the insula for the perception (observation, listening) and expression (actions, words) of vitality form strongly suggests the existence of the mirror mechanism for action and speech vitality forms in the dorsocentral insula. This mechanism could allow individuals from one side to understand action and speech vitality forms expressed by others by remapping these action features on their motor schema to prepare an appropriate motor response (Di Cesare et al., 2016a). It is plausible that the affective state of the agent communicated by speech and action vitality forms may modulate the motor behavior of the receiver during social interactions. In order to test this hypothesis, our group carried out a kinematic study (Di Cesare et al., 2017a). Specifically, the participants were presented with stimuli showing a motor request (give me, take it) expressed gently or rudely and presented in visual modality, auditory modality, or mixed (visual and auditory) modality and, according to the request (give me or take it), they were asked to take or give a bottle. Results indicated that the vitality forms of the request (gentle or rude) influenced the execution of actions performed by the receiver. In particular, when participants perceived a rude request, they interacted with the object with a larger trajectory and a higher velocity. Whereas a gentle request produced a kind interaction with the object, corresponding to a smaller trajectory and a lower velocity. These findings represent first evidence that vitality forms expressed by an agent affect the motor behavior of the receiver.

Since human interactions are characterized by the expression and the perception of vitality forms, the next step is to understand whether, besides the execution of actions, vitality forms may also affect the perception of actions. Indeed, since perception, planning, and execution of action and speech vitality forms are based on the same neural circuit, in the present study, we hypothesized that the vitality form of a motor request (give me) expressed vocally may influence the internal representation of a subsequent action (passing an object) by modifying some features, such as its time duration.

In order to address this issue, we carried out two behavioral experiments aiming to: (1) measure how gentle and rude vitality forms may affect the way of perceiving actions during an action observation task (experiment 1), and (2) quantify the duration of this effect (experiment 2). In the first experiment, participants were required to perform a cognitive task. Specifically, they listened to a voice of an actor and an actress pronouncing a motor request “give me” (“dammi”: Italian verb) in a rude or gentle way. After listening to the vocal request, participants observed video clips showing only the initial part of a rude or gentle action (passing an object) and were required to continue mentally the action, indicating the time of its end. Leveraging on the task used for this first experiment, time delays of different durations were added between the vocal request and the action presentation in the second experiment.

In line with our hypothesis, results showed that listening to a gentle vocal request increased the estimated duration of an action (passing an object) subsequently presented. In contrast, listening to a rude vocal request decreased the estimated duration of the same action. This effect lasted 800 ms and then started to decay.

## Materials and Methods

### First Experiment

#### Participants

Thirty healthy right-handed volunteer subjects (20 women and 10 men, mean age = 24.4; SD = 2.87) participated in this study. All the participants had normal or corrected to normal visual acuity. Nobody reported a history of psychiatric or neurological disorders or current use of psychoactive drugs. This behavioral study was approved by the ethics committee of the University of Parma (UNIPRMR750v1) in accordance with the Declaration of Helsinki as preliminary to fMRI experiments.

#### Visual Stimuli

**FIGURE 1:**
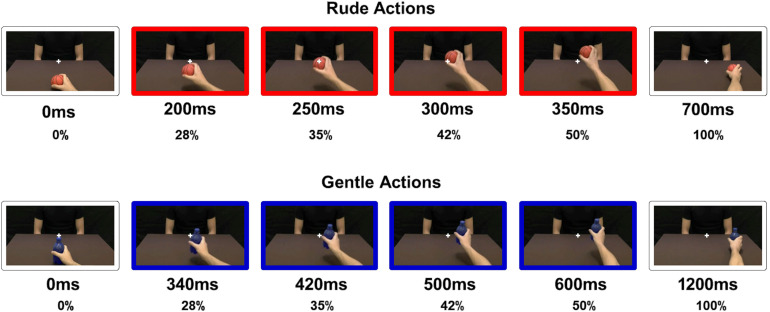
Video stimuli presented to participants. For rude actions (red color): 200, 250, 300, or 350 ms, corresponding to 28, 35, 42, and 50%, respectively, of the total duration (700 ms). For gentle actions (blue color): 340, 420, 500, or 600 ms, corresponding to 28, 35, 42, and 50%, respectively, of the total duration (1,200 ms).

Participants sat comfortably in front of a table on which a laptop was placed, positioning their right hand on the mouse. The stimuli consisted of video clips showing an actor passing different objects (a packet of crackers, a ball, a bottle, or a cup) to another person in a gentle or a rude way. Particularly, half of the passing actions were performed towards another male actor and the other half towards a female actress. It is important to note that, in order to facilitate the motor representation of the observed action in participants, video clips showed actions performed by a right hand with an egocentric perspective (Rizzolatti et al., 2021). Specifically, videos were obscured so that participants observed only a portion of the entire duration (Figure 1). Stimuli presented could last 28, 35, 42, and 50% of the total duration of action. For rude actions (total duration: 700 ms), stimuli could last 200, 250, 300, or 350 ms. For gentle actions (total duration: 1,200 ms), stimuli could last 340, 420, 500, or 600 ms. Stimuli have been presented using E-Prime software in a random order.

#### Task and Experimental Paradigm

**FIGURE 2:**
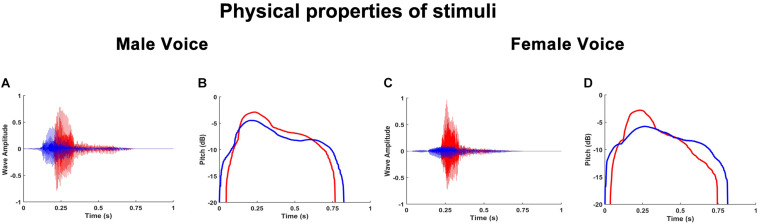
Graphs show the wave amplitude of man **(A)** and woman **(C)** voices; the pitch of man **(B)** and woman **(D)** voices. Red color refers to rude vitality form and blue color refers to gentle vitality form.

During the experiment, participants were stimulated with a vocal request consisting of a voice of an actor/actress, pronouncing “give me” (Italian verb: “dammi”) in a rude or gentle way. Half of the vocal requests were performed by an actress and the other half by an actor. After each vocal request, participants were presented with the video stimuli described above and were required to continue the partially observed action mentally and to estimate the time of its end, by pressing the mouse button. Each vocal request was recorded by using a condenser microphone (RODE NT1) placed 30 cm away in front of the actors and digitized with a phantom-powered A/D converter module (M-AUDIO M-TRACK). After recording, the audio files were processed with COOL EDIT PRO software to obtain the final version of the stimuli. Rude and gentle vocal requests differed for parameters such as the wave amplitude (Figures 2A,C) and the pitch (Figures 2B,D).

**FIGURE 3:**
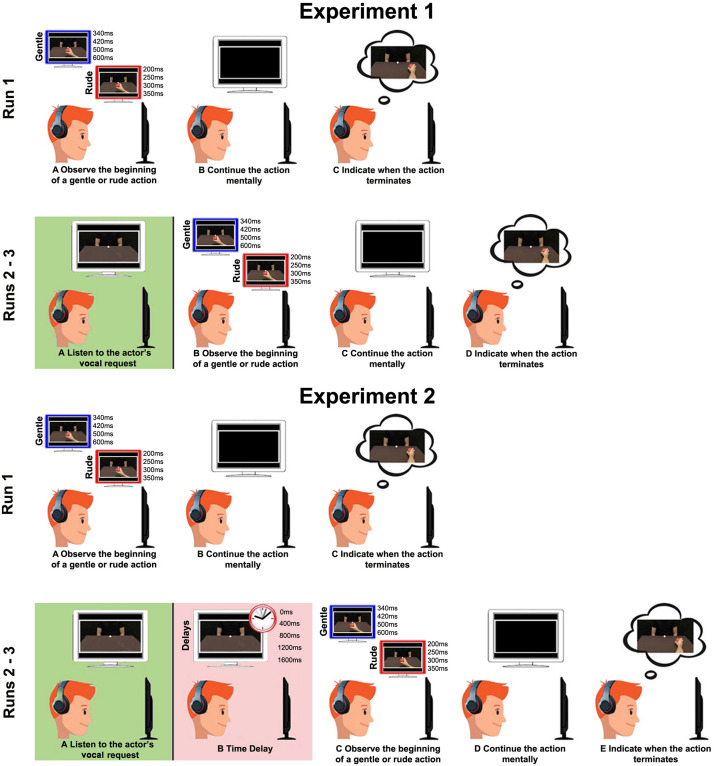
For both the experiments, in Run 1: **(A)** participants observed the beginning of a gentle or rude action; **(B)** continued the action mentally; and **(C)** indicated the time of its end. For Experiment 1, in Run 2 and Run 3: **(A)** participants listened to a rude/gentle vocal request; **(B)** observed the beginning of the same gentle or rude action; **(C)** continued the action mentally; and **(D)** indicated the time of its end. In Experiment 2, Run 2 and Run 3 were the same and participants were required to perform the same task **(E)**, but one of four possible time delays was inserted between the vocal request and the video stimuli (400, 800, 1200, and 1600 ms).

Four conditions were randomly presented by using E-Prime software: (1) RDV_RDA (rude vocal and rude action: congruent condition): the vocal request and the observed action were both rude; (2) GTV_GTA (gentle vocal and gentle action: congruent condition): the vocal request and the observed action were both gentle; (3) RDV_GTA (rude vocal and gentle action: incongruent condition): the vocal request was rude and the observed action was gentle; and (4) GTV_RDA (gentle vocal and rude action: incongruent condition): the vocal request was gentle and the observed action was rude. Each video was presented seven times for each condition. Before the beginning of the experiment, participants were asked to perform a training session to become familiar with the experimental task. In particular, first, they were presented with video clips showing the entire duration of the action and subsequently were required to observe the initial part of the same action and estimate the time of its completion. The experiment was composed of three different runs (Figure 3). In the first run, the participants simply performed the task, without receiving vocal requests before, to assess their capacity to correctly estimate the duration of rude and gentle actions. This run was used as a baseline and lasted 2 min. In the second and third runs, participants listened to the vocal request, expressed with rude or gentle vitality form, and then they observed the beginning of the action and estimated the time of its end. The duration of the second and third runs was 9 min.

## Second Experiment

### Participants

Thirty-eight healthy right-handed volunteer participants (21 women and 17 men: mean age = 24.4; SD = 4) took part in this study. All participants had normal or corrected to normal visual acuity. Nobody reported a history of psychiatric or neurological disorders or current use of psychoactive drugs. This behavioral study was approved by the ethics committee of the University of Parma (UNIPRMR750v1) in accordance with the Declaration of Helsinki as preliminary to fMRI experiments.

### Task and Experimental Paradigm

In the second experiment, participants were only presented with video clips of 200 ms for rude actions and 340 ms for gentle actions, i.e., the shortest among the stimuli described above (28% of action duration). The task they were asked to perform was the same as that of the first experiment: they listened to the vocal request expressed gently or rudely by the actor or actress, and then they observed the initial part of the action and estimated the time of its completion by pressing the mouse button. In order to estimate the duration of the effect of vocal request conveying vitality forms on the task performed afterward, time delays were added between the vocal request and the presentation of video clips.

Time delays consisted of 0 ms (meaning no delay between vocal request and video stimuli, as in the first experiment), 400, 800, 1,200, or 1,600 ms. The second experiment was composed of three runs, and stimuli were presented randomly by using E-Prime software (Figure 3). For both the experiments, responses of the participants were modeled by using mixed linear models (MLMs), with time delay and experimental condition as fixed effects. In order to detect the best fit, we compared models containing only fixed effects, random intercept or fixed slopes, both random intercept and fixed slopes by using Akaike’s Corrected Criterion (AICc) as a selection criterion. For both experiments, the models with random intercept and slopes were the best. The significance level was fixed at *p* = 0.05. All *post hoc* analyses used the Benjamini–Hochberg procedure (False Discovery Rate) to control Type I errors in multiple pairwise comparisons.

## Results

### First Experiment

**FIGURE 4:**
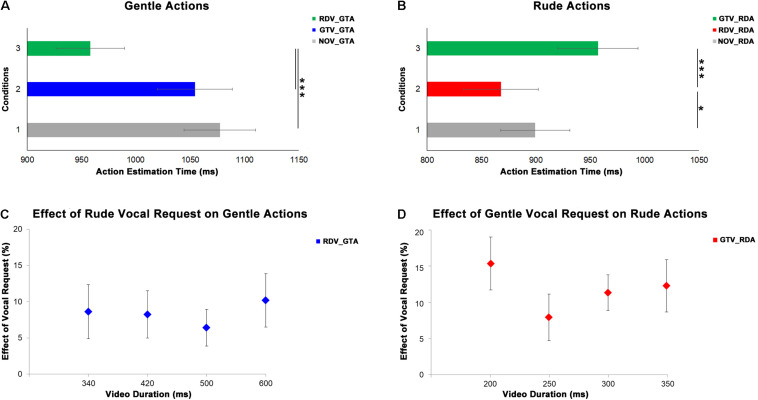
At the top, graphs show the results obtained from the analysis of gentle **(A)** and rude **(B)** actions estimation. Gray bars refer to the baseline condition (no vocal request: NOV). Green bars refer to incongruent conditions: rude vocal request and gentle action (RDV_GTA) in panel **(A)**, gentle vocal request and rude action (GTV_RDA) in panel **(B)**. Blue bars in panel **(A)** refer to gentle congruent condition: gentle vocal request and gentle action (GTV_GTA). Red bars in panel **(B)** refer to rude congruent condition: rude vocal request and rude action (RDV_RDA). At the bottom, graphs show the effect of rude vocal requests on gentle action estimation **(C)** for four different durations (340, 420, 500, and 600 ms) and the effect of gentle vocal requests on rude action estimation **(D)** for four different durations (200, 250, 300, and 350 ms). *p < 0.05, and ***p < 0.001.

In order to evaluate the effect of vocal requests conveying gentle and rude vitality forms on the perception of observed actions, we analyzed the responses of the participants (estimated action durations). The first MLM comprised the estimated durations of four video clips (340, 420, 500, and 600 ms) of the participants showing gentle actions in three experimental conditions (baseline: NOV_GTA, no vocal and gentle action; congruent: GTV_GTA; incongruent: RDV_GTA). The second MLM comprised the estimated durations of four video clips (200, 250, 300, and 350 ms) of the participants showing rude actions in three experimental conditions (baseline: NOV_RDA, no vocal and rude action; congruent: RDV_RDA, incongruent: GTV_RDA). Results of the first MLM analysis indicated a significant principal effect of experimental conditions (*F*_[__2__;__319__]_ = 11.67, *p* < 0.0001) and durations (*F*_[__3__;__319__]_ = 3.81, *p* = 0.011; Figure 4A); their interaction was very close the significance level (*F*_[__6__;__319__)_ = 1.94, *p* = 0.073). *Post hoc* analyses revealed a significant difference between incongruent condition and both baseline (*p* < 0.001) and congruent (*p* = 0.001) conditions; significant differences between 340 ms delay and both 420 (*p* = 0.035) and 500 ms (*p* = 0.034); interaction (340 ms: NOV_GTA vs. RDV_GTA, *p* < 0.001, GTV_GTA vs. RDV_GTA, *p* < 0.001; 420 ms: NOV_GTA vs. RDV_GTA, *p* < 0.05, GTV_GTA vs. RDV_GTA, *p* < 0.01; 500 ms: NOV_GTA vs. RDV_GTA, *p* < 0.05, GTV_GTA vs. RDV_GTA, *p* < 0.05; 600 ms: NOV_GTA vs. RDV_GTA, *p* < 0.001, GTV_GTA vs. RDV_GTA, *p* < 0.01).

Results of the second MLM analysis indicated a significant difference between experimental conditions (*F*_[__2__;__319__]_ = 10.86, *p* < 0.001; Figure 4B), between durations (*F*_[__3__;__319__]_ = 21.22, *p* = 0.0001) and again the interaction close to the significance level (*F*_[__6__;__319__]_ = 1.99, *p* = 0.066). *Post hoc* analyses showed: a significant difference between congruent condition and both baseline (*p* = 0.021) and incongruent (*p* < 0.001) conditions; significant differences between 200 ms delay and all others delays (250 ms: *p* = 0.001; 300: *p* < 0.001; 350: *p* = 0.001) as well as between 250 and 350 ms (*p* < 0.001) and between 300 and 350 ms (*p* = 0.004) interaction. For each duration, *post hoc* analysis revealed a significant difference among experimental conditions (200 ms: NOV_RDA vs. RDV_RDA, *p* < 0.05, RDV_RDA vs. GTV_RDA, *p* < 0.001; 250 ms: RDV_RDA vs. GTV_RDA, *p* < 0.07; 300 ms: NOV_RDA vs. GTV_RDA, *p* < 0.05, RDV_RDA vs. GTV_RDA, *p* < 0.001; 350 ms: NOV_RDA vs. GTV_RDA, *p* < 0.05, RDV_RDA vs. GTV_RDA, *p* < 0.01. In order to quantify the effect of vocal requests on the action estimation for each duration, we compared values obtained in congruent conditions with those obtained in incongruent conditions [| (RDV_GTA – GTV_GTA)| ^∗^100/GTV_GTA for gentle actions; | (GTV_RDA – RDV_RDA)| ^∗^100/RDV_RDA for rude actions]. Then two MLM were used to assess possible differences of effects of vocal requests on the actions durations. The first MLM was used for the gentle actions while the second MLM model was used for the rude ones. Results of this analysis revealed no differences among action durations (*p* > 0.05) showing that the effect of vocal request on estimated action duration for both gentle and rude actions was not sensible to different time durations (Figures 4C,D).

### Second Experiment

**FIGURE 5:**
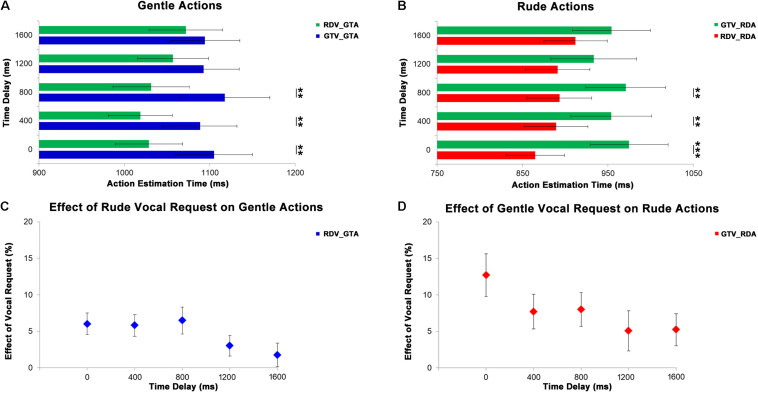
At the top, graphs show results obtained from the analysis of gentle **(A)** and rude **(B)** actions estimation. Green bars refer to incongruent conditions: rude vocal request and gentle action (RDV_GTA) in panel **(A)**, gentle vocal request and rude action (GTV_RDA) in panel **(B)**. Blue bars in panel **(A)** refer to gentle congruent condition: gentle vocal request and gentle action (GTV_GTA). Red bars in panel **(B)** refer to rude congruent condition: rude vocal request and rude action (RDV_RDA). At the bottom, graphs show the effect of rude vocal requests on gentle action estimation **(C)** and the effect of gentle vocal requests on rude action estimation **(D)** for five different time delays (0, 400, 800, 1,200, and 1,600 ms). **p < 0.01, and ***p < 0.001.

In order to evaluate how long the effect of vocal requests conveying different vitality forms last, we analyzed the responses (estimated action durations) of the participants after different delays were interposed between the request and the perception of action. The first MLM comprised the participant’ estimated durations of four video clips showing gentle actions after five different time delays (0, 400, 800, 1200, 1600 ms) in two experimental conditions (congruent: GTV_GTA, incongruent: RDV_GTA). The second MLM comprised the participant’ estimated durations of four video clips showing rude actions after five different time delays (0, 400, 800, 1200, 1600 ms) in two experimental conditions (congruent: RDV_RDA, incongruent: GTV_RDA). Results of the first MLM analysis indicated the condition principal effect (*F*_[__1__;__333__]_ = 16.91, *p* = 0.001) and the interaction between experimental conditions and time delays (F_[__4__;__333__]_ = 3.06, *p* = 0.017) as significant, whereas the time delays principal effect was only close to the significance level (*F*_[__4__;__333__]_ = 2.14, *p* = 0.076). *Post hoc* analysis revealed a significant difference among experimental conditions for 0 ms (*p* = 0.021), 400 ms (*p* = 0.023), and 800 ms (*p* = 0.019) time delays, but not for time delays of 1200 ms (*p* = 0.086) and 1600 ms (*p* = 0.318; Figure 5A).

The second MLM showed a significant difference among experimental conditions (*F*_[__1__;__333__]_ = 14.21, *p* < 0.001) and a significant interaction (*F*_[__4__;__333__]_ = 3.39, *p* = 0.01), but no effect for time delay (*F*_[__1__;__333__]_ = 1.68, *p* = 0.155). *Post hoc* tests showed a similar pattern of comparisons to that observed in the previous analysis: a significant difference among experimental conditions for 0 ms (*p* < 0.001), 400 ms (*p* = 0.009), and 800 ms (*p* = 0.005) time delays, but not for time delays of 1200 ms (*p* = 0.132) and 1600 ms (*p* = 0.170; Figure 5B). As in the first experiment, in order to quantify the effect of vocal requests on the estimation of duration of action for each time delay, we compared values obtained in congruent conditions with those obtained in incongruent conditions by using an MLM model for the gentle action and an MLM for the rude ones. Results of this analysis revealed no differences in the vocal request effect for both gentle and rude actions among the action time delays (*p* > 0.05) (Figures 5C,D).

### Overall Effect of Vocal Requests on Action Estimation

**FIGURE 6:**
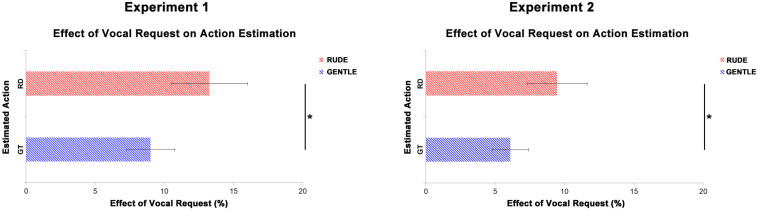
Overall effect of gentle vocal requests on the estimation of rude (RD, red bars) actions duration and the overall effect of rude vocal requests on the estimation of gentle (GT, blue bars) actions duration. Graphs report standard errors (SE). * indicate statistical significance (*p* < 0.05) in a paired sample *t*-test.

Figure 6 shows the overall effect of vocal requests conveying gentle and rude vitality forms on the estimation of action duration. For the first experiment, we averaged values obtained from the comparison between congruent and incongruent conditions for four durations (gentle: 340, 420, 500, 600 ms; rude: 200, 250, 300, 350 ms). For the second experiment, we averaged values obtained from the comparison between congruent and incongruent conditions for the three significant time delays (0, 400, and 800 ms). Then two paired sample *t*-tests were carried out to assess possible differences between the effect on gentle action estimation (rude vocal request) and on rude action estimation (gentle vocal request). For both experiments, results showed a significant difference between rude and gentle vitality form (Experiment 1: *t*(29) = 5.2, *p* = 0.001; Experiment 2: *t*(37) = 4.6, *p* = 0.0001). Specifically, the effect of gentle vocal requests on rude action estimation was greater than the effect of rude vocal requests on gentle action estimation.

## Discussion

By observing actions, people may understand two different components: their goal and their form. The goal represents *what* someone is doing while the form represents *the manner* in which the action is performed. The form of an action has a strong influence on the interaction between humans. According to their affective state, the agent may perform the same gesture with different forms [named vitality forms by Daniel Stern (2010)] expressing positive or negative attitudes toward the receiver (Di Cesare et al., 2020b). Although humans continuously convey vitality forms through actions, sounds, speech, and touches, to date the impact of vitality forms expressed by an agent on the action perception of the receiver has never been investigated. The present study had two main goals: (1) to evaluate how a positive/negative vocal request may affect the estimation of action duration; (2) to measure how long this effect lasts. In the first experiment, the analysis of the ability of the participants to estimate the timing of the end of the action showed that listening to a gentle vocal request (give me) increased the estimated duration of an action (passing an object), subsequently presented. In contrast, listening to a rude vocal request decreased the estimated duration of the same action. Specifically, in the incongruent condition, when the participants listened to a rude voice and then observed the initial part of a gentle action, they anticipated its end. In contrast, if they listened to a gentle voice and then observed the initial part of a rude action, they estimated the action as lasting longer. In the second experiment, results showed that this effect lasted for 800 ms, and then it started to decay. In line with our results, data are provided by a psychophysical study carried out by Lombardi et al. (2021) showing that listening to the same vocal requests or perceiving a physical request both expressed rudely or gently influenced the perception and execution of actions. Altogether, these findings suggest that vitality forms expressed vocally by an agent automatically influence the perception of a subsequent action observed by the receiver. An interesting question is to understand how it is possible. When individuals observe actions performed by others, they are able to understand the goals of the action as well as their intentions. These abilities are related to the existence of a basic brain mechanism known as “mirror mechanism” based on the activity of a set of neurons located in parietal and frontal areas that discharge both when individuals perform a goal-directed action and when individuals observe another person performing the same action. In the last few years, besides the action goal, research has been carried out to identify the neural mechanisms underlying the ability to processing how actions are performed, i.e., the vitality form. In a series of fMRI studies (Di Cesare et al., 2015, 2016a,2016b,2017b, 2019; Di Cesare, 2020; Di Cesare et al., 2020a,b; Rizzolatti et al., 2021). Di Cesare and colleagues found that the perception and the expression of different vitality forms activate the dorsocentral insula. Indeed, the perception of action, speech, and touches conveying vitality forms activate the dorsocentral insula, more specifically the middle and posterior insula short gyri. Interestingly, the same insular sector is also active during the expression and imaging of actions and speech expressing the same vitality forms (rude, gentle). The authors showed that the dorsocentral insula is the key region encoding vitality forms, and that it is also endowed with a mirror mechanism that makes the decoding of the vitality forms of others possible. Differently from the mirror mechanism located in the parietal and frontal areas, specific for action goal understanding (Rizzolatti and Craighero, 2004; Iacoboni and Dapretto, 2006; Fabbri-Destro and Rizzolatti, 2008; Keysers and Fadiga, 2008; Caspers et al., 2010; Grosbras et al., 2012; Molenberghs et al., 2012; Rizzolatti and Sinigaglia, 2016), the mirror mechanism located in the insula might allow one to express their own mood/attitude and to understand those of others (Di Cesare et al., 2020b). In this view, it is plausible that the dorsocentral insula transforms the vitality-form information of the vocal request into a motor domain, allowing participants from one side to understand vitality forms expressed by the actor from the other to prepare an adequate response to a subsequent action. This mechanism, fundamental for social communication, would allow individuals to obtain a fluidity of interaction that characterizes our everyday encounters with others.

One may hypothesize that the affective contagion of the vocal request on the action perception may be ascribed to a potential arousal effect. Specifically, it is plausible that listening to a rude voice conveying an imperative request may induce the receiver to assume an alert state, making his action response faster. However, this putative effect is not in line with results concerning the estimation of duration of gentle actions. Although the participants were required to estimate the duration of rude actions, the gentle vocal request induced them to become slower, estimating the actions as lasting longer. Moreover, in order to better clarify this point, we carried out a further analysis showing that the effect of gentle vocal request on rude action estimation was significantly greater than the effect of rude vocal request on gentle action estimation in both the experiments. This suggests that the influence of vitality forms on action estimation was not merely due to an arousal effect, because it was easier to slow down the response of the participants than to speed it up. It is important to point out that we considered a vocal request consisting in one simple imperative action verb “give me,” pronounced by man or woman actors in a rude or gentle way. This limitation may be overcome in the future by reproducing a more realistic dialogue conveying vitality forms. It is plausible that in this way the effect found in the present study may have a longer duration overtime, affecting behavior of the participants in a stronger way.

Findings provided in the current study regarding the influence of vitality forms on the perception of the action contribute to extending the knowledge on the role of the affective states on the automatic contagion effect of others. Previous studies showed an affective convergence during facial mimicry (Dimberg, 1982; Dimberg et al., 2000; Dimberg and Thunberg, 2012; Varcin et al., 2019), body postures (Schmidt et al., 2011), and vocalizations (Cappella and Planalp, 1981; Fujiwara and Daibo, 2016), supporting the existence of an automatic mechanism selective for the affective contagion. In a recent study (Pinilla et al., 2020), Pinilla and colleagues demonstrated that when participants were induced to a negative affective state, they judged both angry and happy faces closer to a negative affective state. In contrast, when participants were induced to a positive affective state, they judged both the happy and angry faces closer to a positive affective state.

Moreover, our study represents the first demonstration that by observing a small part of a goal-directed action, besides the goal, the observer is also able to understand the vitality form of the action. It is plausible that, during the task, participants may have “read” the partial kinematic information of the action and remapped it on their own motor repertoire. This perception–action remapping would have allowed them to simulate internally the vitality forms of action (Jeannerod and Decety, 1995).

In conclusion, our study provides three main findings. First, we demonstrated that vitality forms conveyed by vocal requests influence the perception of actions of participants. Second, this contagion effect lasts 800 ms and then starts to decay. Finally, we provide first evidence that by observing a goal-directed action, besides the goal, the observer is able to internally simulate the vitality forms of that action.

## Data Availability Statement

The original contributions presented in the study are included in the article/supplementary material, further inquiries can be directed to the corresponding author/s.

## Ethics Statement

The studies involving human participants were reviewed and approved by University of Parma. The patients/participants provided their written informed consent to participate in this study.

## Author Contributions

GDC designed the research. GDC and SA performed the experiment. GDC and AP analyzed the data. AS, AP, and GL contributed toward material and consulting for the development of the project. GDC, AP, GL, and AS wrote the first draft. All authors read the manuscript and contributed to its final form.

## Conflict of Interest

The authors declare that the research was conducted in the absence of any commercial or financial relationships that could be construed as a potential conflict of interest.

## Publisher’s Note

All claims expressed in this article are solely those of the authors and do not necessarily represent those of their affiliated organizations, or those of the publisher, the editors and the reviewers. Any product that may be evaluated in this article, or claim that may be made by its manufacturer, is not guaranteed or endorsed by the publisher.
